# Supplementary material for: Differences and Associations of NLRP3 Inflammasome Levels with Interleukins 1α, 1β, 33 and 37 in Adults with Prediabetes and Type 2 Diabetes Mellitus
Source: Biomedicines. 2023 Apr 28;11(5):1315. doi: 10.3390/biomedicines11051315 (PMC10216290; doi:10.3390/biomedicines11051315)
Supplement: Supplementary file 1 [file biomedicines-11-01315-s001.zip › biomedicines-2347729-supplementary.pdf]

Supplementary Data:

Table S1. Correlation analysis for Log-NLRP3 with other parameters ILs in different groups (HC, PD, T2DM).

Parameters	Overall	HC	PD	T2DM
IL-1α#	0	−0.18 *	0.45 **	0.12
IL-1β#	0	−0.12	0.03	0.35 *
1l-33#	0.38 **	0.41**	0.38 **	0.27 *
IL-37#	0.14 *	0.11	0.19	0.19

Note: Data presented (R) co-efficient. # represent Log-transformed variables. * and ** denote *p*-values at 0.05 and 0.01 level, respectively.

Table S2. Correlation analysis for Log-NLRP3 with other parameters and ILs in males and females.

Parameters	Overall	Males	Females
IL-1α#	0	−0.12	0.16 *
IL-1β#	0	0.15	−0.08
1l-33#	0.38 **	0.46 **	0.24 **
IL-37#	0.14 *	0.02	0.16 *

Note: Data presented (R) co-efficient. # represented Log-transformed variables. * and ** denote *p*-values at 0.05 and 0.01 level, respectively.
